# Platypnea-Orthodeoxia Syndrome From Atrial Septal Defects: A Rare Primum Case and Literature Review

**DOI:** 10.7759/cureus.90062

**Published:** 2025-08-14

**Authors:** Areeb Ahmad, Pritham S Pinni, Mohamad Labban, Shreya Mishra, Almatmed Abdelsalam

**Affiliations:** 1 Internal Medicine, University of Central Florida College of Medicine, Orlando, USA; 2 College of Medicine, University of Central Florida, Orlando, USA; 3 Cardiology, Health First, Cape Canaveral, USA; 4 Internal Medicine, Health First, Cape Canaveral, USA

**Keywords:** atrial septal defect (asd), intracardiac shunt, platypnea-orthodeoxia syndrome, primum asd, upright hypoxemia

## Abstract

Platypnea-orthodeoxia syndrome (POS) is characterized by hypoxemia that worsens in the upright position and improves when lying down. While various causes exist, most cases involve intracardiac shunting through a patent foramen ovale, whereas atrial septal defects (ASDs) are less common and present in diverse embryological forms. Most documented ASD-associated cases involve the secundum type, whereas the primum subtype remains notably rare in the literature. We report a rare case of POS caused by a primum ASD in a 68-year-old female. Transesophageal echocardiography was essential for locating the primum ASD, which facilitated successful percutaneous closure and complete resolution of her symptoms. Additionally, we reviewed 18 published cases of ASD-associated POS to better characterize the presentation, anatomical contributors, and treatment outcomes of this cause.

## Introduction

Platypnea-orthodeoxia syndrome (POS) is a rare, often overlooked condition defined by positional dyspnea and hypoxemia that worsen upon sitting or standing and improve when lying down. While it can arise from a variety of cardiopulmonary anomalies, approximately 87% of cases involve intracardiac shunting through a patent foramen ovale (PFO), atrial septal defect (ASD), or fenestrated atrial septal aneurysm (ASA) [1]. The remaining cases usually arise from intrapulmonary shunting due to ventilation/perfusion mismatch, pulmonary arteriovenous malformations, or hepatopulmonary syndrome [2].

Due to its obscure presentation, POS is frequently misdiagnosed or attributed to more common causes of hypoxemia such as pulmonary embolism, pneumonia, chronic obstructive pulmonary disease (COPD), interstitial lung disease, or heart failure [3]. In such cases, observing positional oxygen desaturation can serve as a critical diagnostic clue, prompting further evaluation with bubble contrast transthoracic echocardiography and, when necessary, transesophageal echocardiography (TEE) for detailed assessment of interatrial anatomy and shunt dynamics [1].

Although the role of PFO in POS has been extensively studied, with PFOs accounting for 67% of all shunt types, ASDs are less common (12%) and present in various embryological forms [1,3,4]. Most ASD cases reported in the literature are of the secundum type, with other types, including primum ASDs, rarely documented. In this case report, we highlight a rare case of primum ASD-associated POS, contributing new insights into this uncommon variant. Additionally, we systematically examine the published literature on POS caused exclusively by all ASD types to better define the clinical patterns, diagnostic pathways, and treatment strategies in this subgroup.

## Case presentation

A 68-year-old woman with a past medical history of anxiety disorder and breast cancer (status post lumpectomy and chemotherapy in 2017) presented to Cape Canaveral Hospital with progressive shortness of breath and fatigue over several weeks. She was scheduled for a routine colonoscopy as part of standard cancer surveillance, but the procedure was deferred due to acute respiratory distress. She was referred to the emergency department (ED) for further evaluation.

**Figure 1:**
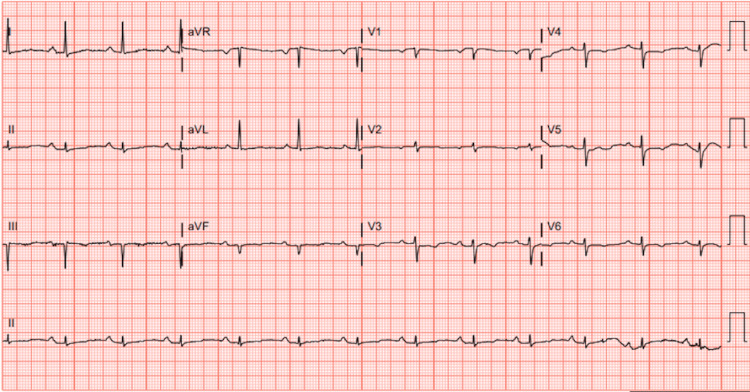
Electrocardiogram demonstrating normal sinus rhythm without ST-segment elevations, depressions, or T-wave inversions.

Upon arrival, the patient was in moderate respiratory distress, requiring five liters of supplemental oxygen via nasal cannula. Physical examination revealed tachypnea but was otherwise unremarkable. Initial testing showed normal troponin and B-type natriuretic peptide (BNP) levels. Electrocardiography showed normal sinus rhythm without ST-segment abnormalities but with a prolonged QTc interval of 531 ms (Figure 1). 

**Figure 2:**
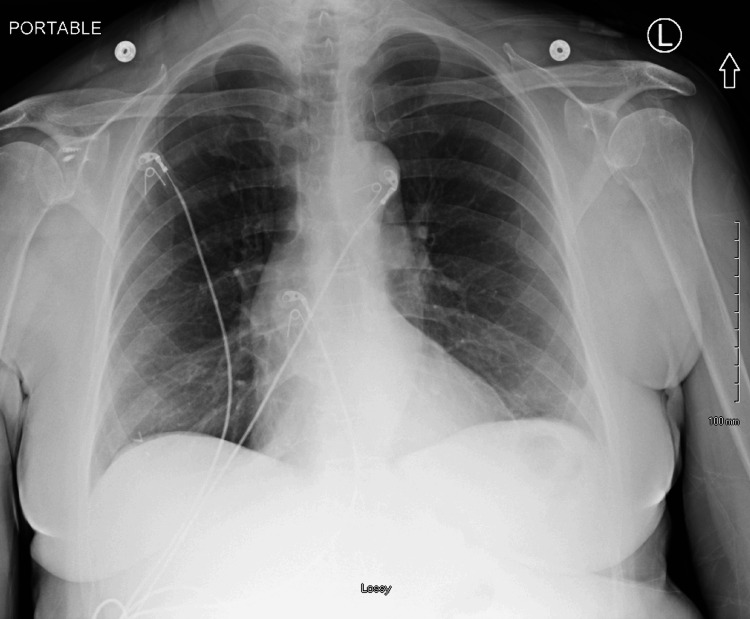
Portable AP Chest X-ray showing no acute pulmonary findings. Mild cardiomegaly is noted.

A portable chest X-ray demonstrated cardiomegaly without evidence of pulmonary edema, pleural effusion, or pneumothorax (Figure 2).

**Figure 3:**
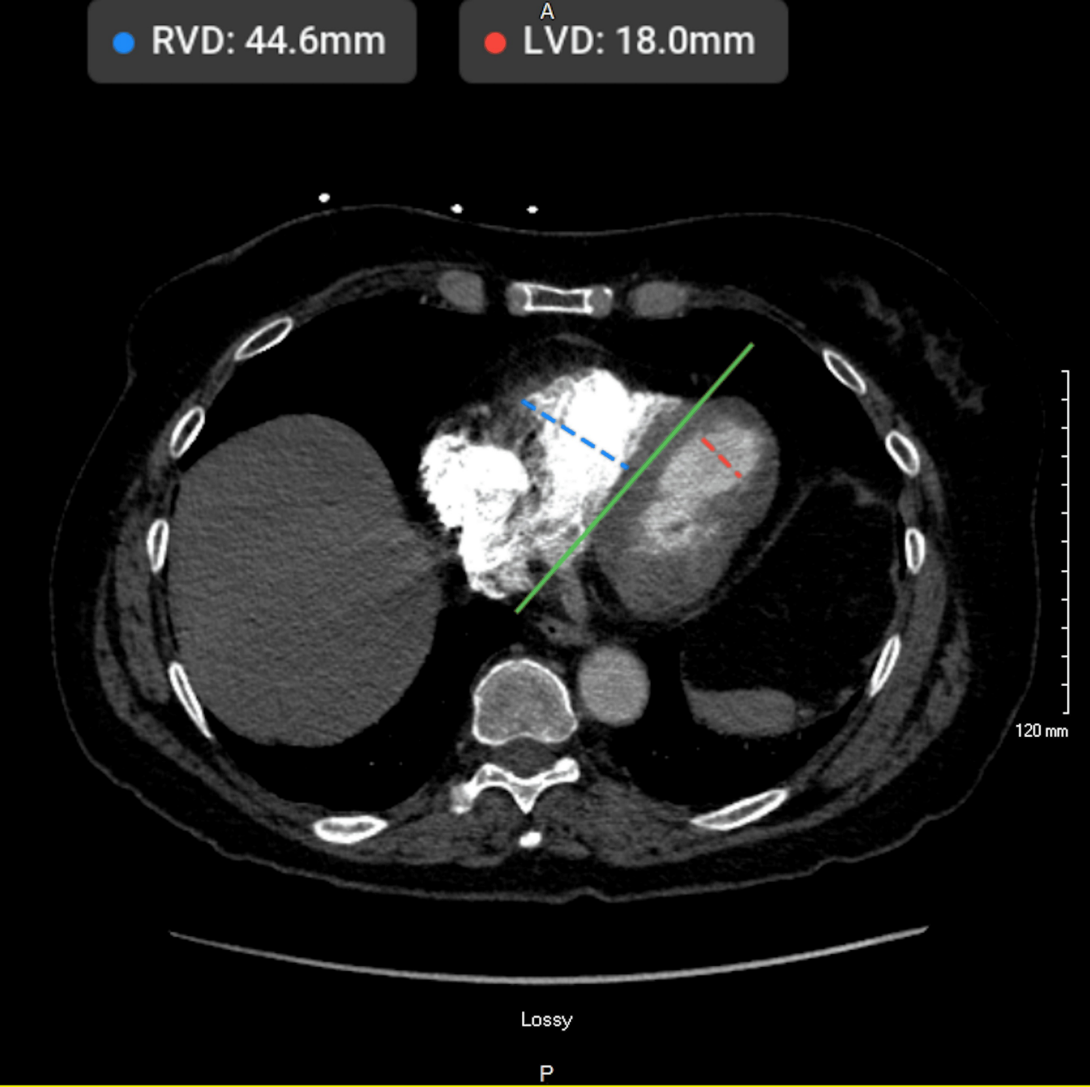
CTA chest showing a dilated right ventricle (blue dashed line, 44.6 mm) and a smaller left ventricle (red dashed line, 18.0 mm). The green line marks the interventricular septum. CTA: computed tomography angiography.

Computed tomography angiography (CTA) of the chest ruled out pulmonary embolism. However, the scan revealed right heart enlargement concerning for strain. A representative axial slice showed a right ventricle (RV) diameter of 44.6 mm and a left ventricle (LV) diameter of 18.0 mm, with a markedly enlarged RV relative to the LV (Figure 3).

**Figure 4:**
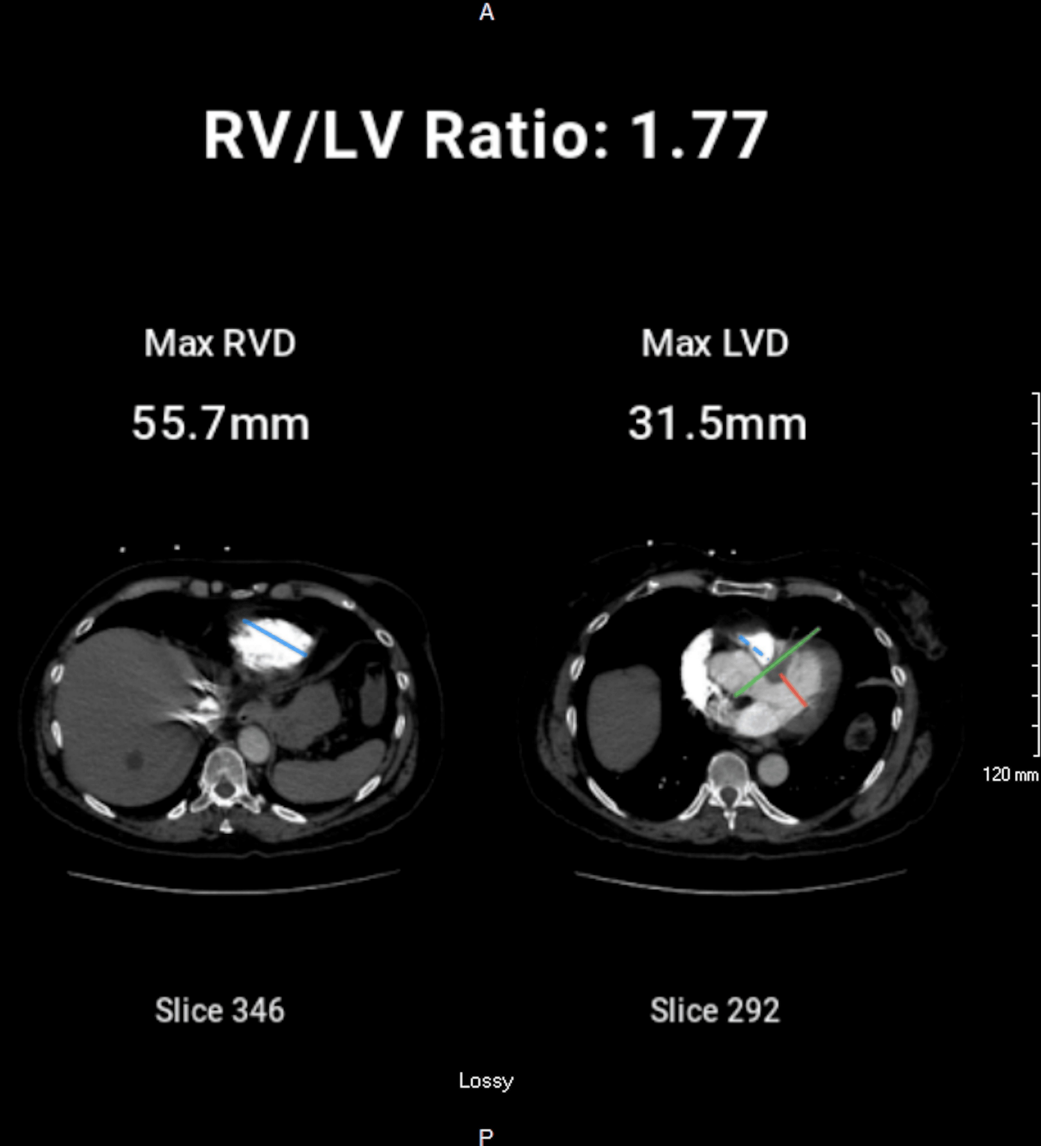
Maximal ventricular diameters on CTA. Left panel (Slice 346): Blue line indicates the maximal transverse diameter of the right ventricle (55.7 mm). Right panel (Slice 292): Red line shows the maximal transverse diameter of the left ventricle (31.5 mm), blue line marks the corresponding right ventricular diameter in the same slice, and the green line denotes the interventricular septum. The resulting RV/LV ratio of 1.77 reflects significant right ventricular enlargement. CTA: computed tomography angiography.

Separate axial images capturing the maximal diameters of each ventricle demonstrated an RV measurement of 55.7 mm and an LV measurement of 31.5 mm, yielding an RV/LV ratio of 1.77 (Figure 4).

A ventilation/perfusion (V/Q) scan also demonstrated low probability for pulmonary embolism, further reducing concern for thromboembolic disease.

Despite these reassuring findings and a partial response to diuretic therapy, which was initiated to assess the possibility of heart failure given the right heart strain, the patient’s oxygen requirement increased to 10 liters. A pivotal observation by the nursing staff revealed that the patient’s oxygen saturation significantly improved when she was placed in the supine position and deteriorated when upright. Cardiology and pulmonology were subsequently consulted due to clinical suspicion of POS.

**Figure 5:**
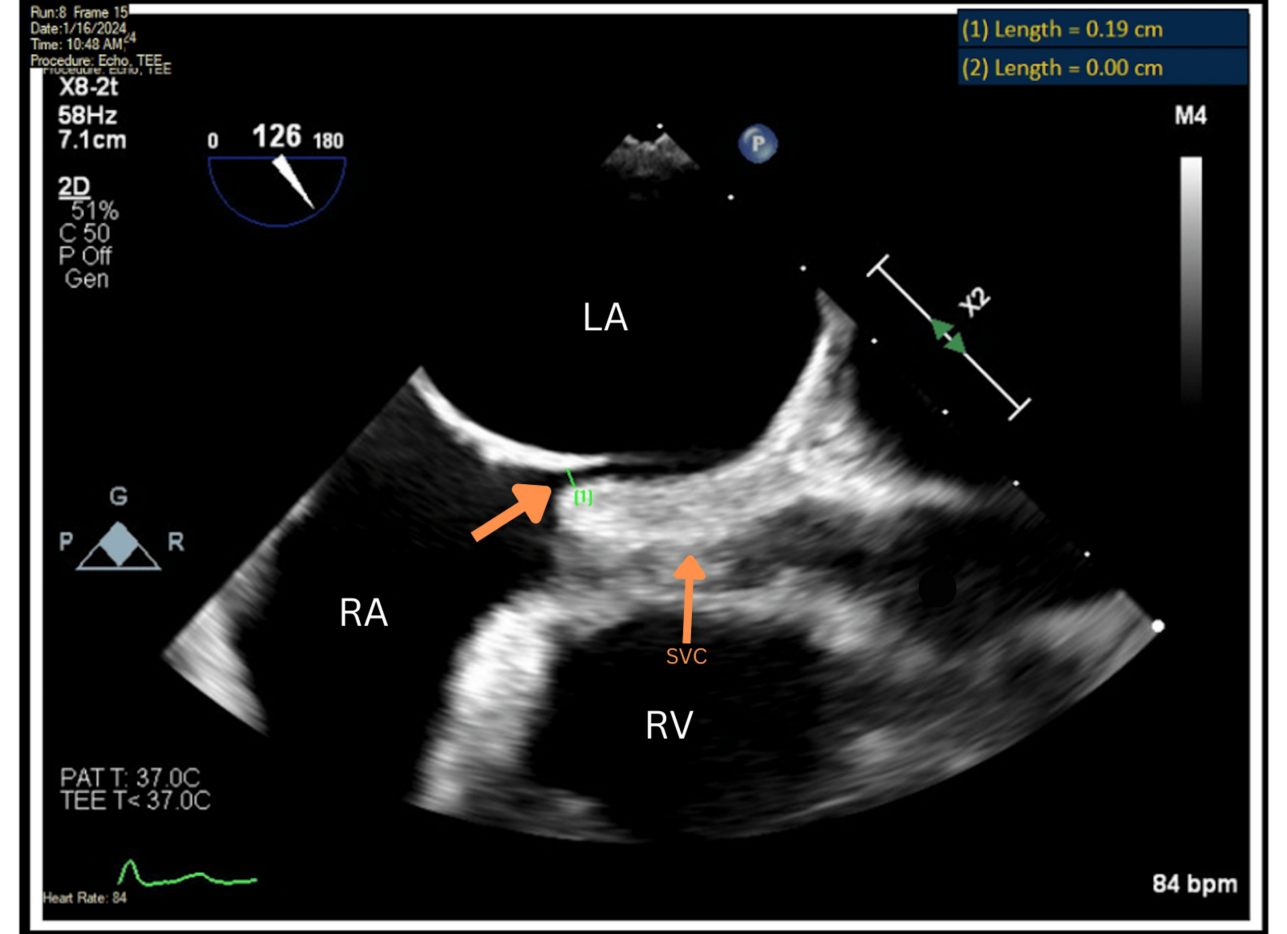
Transesophageal echocardiogram demonstrating a primum ASD. The left orange arrow highlights the measured defect (0.19 cm) between the right atrium (RA) and left atrium (LA). The right orange arrow identifies the superior vena cava (SVC). Cardiac chambers are labeled: RA = right atrium; LA = left atrium; RV = right ventricle. ASD: atrial septal defect.

To assess for an intracardiac shunt, a TEE with agitated saline contrast (bubble study) was performed. This revealed the early appearance of microbubbles in the left atrium, confirming a right-to-left shunt, predominantly in the upright position. A comprehensive TEE was then performed, identifying a small primum ASD measuring 0.19 cm in diameter (Figure 5). 

These findings established the diagnosis of POS secondary to a small primum ASD. The patient was transferred to a tertiary care center, where she underwent successful percutaneous closure of the ASD. Post-procedure, she experienced complete resolution of her dyspnea and oxygen dependence. At follow-up, she had returned to her baseline functional status, including resuming her professional activities as a teacher. She expressed deep appreciation for the clinical team’s persistence and teamwork, which led to the timely diagnosis and resolution of her symptoms. This case emphasizes the value of thorough clinical observation, interdisciplinary collaboration, and maintaining a high index of suspicion for rare but reversible causes of hypoxemia. 

Literature review 

Methodology

We conducted a comprehensive review of the published literature on POS caused exclusively by ASDs. A search on PubMed was performed from inception to April 20, 2025, using the keywords “platypnea orthodeoxia syndrome” and “case report.” The search yielded 264 articles, of which 55 were case reports. Titles and abstracts were screened by AA for relevance, and full texts were reviewed when necessary.

We included only case reports in which the etiology of POS was a true ASD (i.e., secundum, primum, sinus venosus, or unroofed coronary sinus). Cases attributed to PFO, ASA, or intrapulmonary causes (hepatopulmonary syndrome, arteriovenous malformations) were excluded. After screening, 18 articles met the inclusion criteria and were included in the final analysis. Any discrepancies during this phase were resolved by our senior author, AA.

Data extraction was performed by ML using a standardized Excel spreadsheet. The following variables were collected: patient age, sex, relevant past medical history, type of ASD, measurement of the defect, associated anatomical or pathological factors, reported symptoms, primary diagnostic modality, whether postural testing was performed, treatment approach, follow-up duration, and treatment outcome (Table 1).

**Table 1:** ASD-associated POS case characteristics. PAF: paroxysmal atrial fibrillation, V/T/LCF: vertebral/thoracic/lumbar compression fractures, VF: vertebral fracture, POS: platypnea orthodeoxia syndrome, ASD: atrial septal defect, TEE: transesophageal echocardiography, EF: ejection fraction, PMHx: past medical history, DLA/V: dilated left atrium and ventricle, PC: percutaneous closure, AIS: aneurysmal interatrial septum, AGS: agitated saline test, ASO: atrial septal occluder, BS: bubble study, rER: reduced ejection fraction, ASA: atrial septal aneurysm, ICE: intracardiac echocardiogram.

First Author, Year	Journal	Patient Age, Sex	PMHx	Associated Anatomical or Pathological Factors	ASD Type	Measurement	Symptoms Reported	Diagnostic Modality	Procedural Testing Performed (Yes or No)	Treatment Approach	Treatment Outcome
Marzlin et al., 2018 [5]	WMJ	82 F	Nonischemic cardiomyopathy rEF	ASA	Secundum	8 mm	Dyspnea, hypoxemia	TEE with BS	Yes	PC with 30 mm ASO	Eupnea, Normoxic (RA)
Henkin et al., 2015 [6]	Texas Heart Institute	83 F	PAF	Kyphoscoliosis, aortic elongation/dilation	Secundum	8 mm	Dyspnea, hypoxemia	TEE	Yes	PC with 24 mm ASO	Eupnea, Normoxic (RA)
Borgaonkar et al., 2020 [7]	Texas Heart Institute	33 F	Congenital heart block and peripartum cardiomyopathy	Tricuspid regurgitation	Secundum	12 mm	Dyspnea, hypoxemia	ICE and balloon sizing	Yes	PC via 25 mm ASO	Eupnea, Normoxic (RA)
Sanai et al., 2024 [8]	Cureus	67 M	HTN and smoker	ASA	N/A	13 mm	Dyspnea, hypoxemia	TTE with BS	Yes	PC	Eupnea, Normoxia (RA)
Puri et al., 2020 [9]	Cureus	62 M	N/A	N/A	N/A	N/A	Dyspnea, hypoxemia	TEE with BS	Yes	PC	Eupnea, Normoxic (RA)
Abe et al., 2022 [10]	Internal Medicine	81 F	N/A	TVCs	Secundum	18 mm	Dyspnea and hypoxemia	TEE with BS	Yes	PC via 21 mm ASO	Eupnea, Normoxic (RA)
Kazawa et al., 2017 [11]	Internal Medicine	77 F	Brain Abscess	VCF and elongated aorta	Secundum	19 mm	dyspnea, hypoxemia	TTE with BS	Yes	PC	Eupnea, Normoxic (RA)
Ohfuji et al., 2012 [12]	Internal Medicine	79 F	Hysterectomy, TIA, LCF	LCF	N/A	1.5 x0.67 cm	Dyspnea, hypoxemia,	TEE	Yes	PC	Eupnea, Normoxic (RA)
Ugalde et al., 2013 [13]	Journal of Cardiovascular Medicine	73 F	HTN, tobacco use, left lung cancer	N/A	N/A	N/A	Dyspnea, hypoxemia	TEE with contrast injection	Yes	PC	Eupnea, Normoxic (RA)
Asano et al., 2019 [14]	Medicine Circulation Journal	39 F	N/A	Scoliosis, pectum excavatum	N/A	15.4 mm	Dyspnea, hypoxemia	TEE	Yes	PC with 21 mm ASO	Eupnea, Normoxic (RA)
Takiguchi et al., 2013 [15]	Internal Medicine	79F	PE	N/A	Secundum	10.7 x 15.1 mm	Dyspnea, hypoxemia, syncope x2	TEE	Yes	PC	Eupnea, Normoxic (RA)
Bohren et al., 2020 [16]	Case Reports in Critical Care	76 F	HTN, DVT, epidermoid cancer	Right pneumonectomy	N/A	8mm	Respiratory failure with severe hypoxemia	TEE with BS	Yes	PC	Eupnea, Normoxic (RA)
van Gaal et al., 2005 [17]	Cardiovascular Ultrasound	75 F	HTN, Afib	AIS	Secundum	N/A	Dyspnea, hypoxemia	TEE	Yes	PC	Eupnea, Normoxic (RA)
Gomez-Rubin et al., 2012 [18]	Congenital Heart Disease	51 F	Afib, TIA	15 mm Dacron patch dehiscence	secundum	15 mm	Dyspnea and dizziness	TTE with AGS	Yes	Removal of previous device and PC	Eupnea, Normoxic (RA)
Takaya et al., 2014 [19]	Cardiovascular Intervention and Therapeutics	79 F	N/A	LCF	Secundum	8 mm	Dyspnea, hypoxemia	TTE with AGS	Yes	PC via 17 mm ASO	Eupnea, Normoxic (RA)
Lee et al., 2016 [20]	Yonsei Med Journal	20 F	N/A	N/A	Secundum	21.84 mm	Clubbing fingers	TTE with BS	Yes	PC via ASO	Eupnea, Normoxic (RA)
Galiuto et al., 2012 [21]	Journal of Cardiology Cases	65 F	VFs	Dorsal kyphosis, dilated aortic root, VFs	secundum	N/A	Dyspnea, hypoxemia	TEE with AGS	Yes	PC	Eupnea, Normoxic (RA)
Acharya et al., 2000 [22]	Chest	71 M	HTN, retardation	DLA/V	N/A	3x3 cm	Fatigue	TEE	Yes	PC	Eupnea, Normoxic (RA)

Results

A total of 18 case reports of POS secondary to ASDs were included in this review [5-22]. The mean patient age was 66 years (range: 20-83), with a predominance of females (15 females, 3 males).

Associated anatomical or pathological contributors were reported in 17 of 18 cases. The most common categories included spinal deformities or vertebral pathology (compression fractures, scoliosis, kyphosis) in 7 cases, and aortic abnormalities (elongation or dilation of the aorta or aortic root) in 3 cases. Cardiac structural findings such as ASA, tricuspid regurgitation, and chamber dilation were documented in 6 cases. Less frequently noted contributors included prior thoracic surgery, pulmonary pathology, prosthetic patch complications, right ventricular outflow tract reconstruction, and viral infection. One case (6%) reported no associated anatomical findings [15]. Complete case-level details are presented in Table 1.

The anatomical subtype of ASD was identified in 11 of 18 cases (61%). All the classified cases were consistent with a secundum-type ASD. In the remaining 7 cases, the type of ASD was not specified in the original report. Dyspnea and hypoxemia were reported in 16 of 18 cases (89%). The remaining two cases presented with digital clubbing and generalized weakness, respectively [20,22]. Postural oxygen desaturation testing was performed in all 18 cases (100%) and contributed directly to the clinical suspicion and diagnostic workup in each case.

All 18 cases underwent echocardiographic evaluation to identify intracardiac shunting. TEE was performed in 12 cases (67%), of which 8 included bubble contrast using agitated saline. Transthoracic echocardiography (TTE) with bubble study or microbubble contrast was used in 5 cases (28%), and intracardiac echocardiography (ICE) was performed in 1 case (6%).

Definitive closure of the ASD was performed in all 18 cases. Percutaneous closure was performed in 12 cases (67%) using devices such as the Gore Septal Occluder, Gore Helex, Occlutech Figulla Flex II, and Amplatzer Septal Occluder. The remaining 6 patients (33%) underwent surgical closure, including suture repair, patch repair with pericardium, or correction under cardiopulmonary bypass.

## Discussion

Our review highlights a particularly rare etiology within the already uncommon spectrum of POS. Notably, all previously reported cases of ASD-associated POS involved ostium secundum defects, demonstrating a pattern of anatomic consistency in the literature. In contrast, our case documents a primum-type ASD, expanding the existing spectrum of ASD subtypes implicated in POS and contributing a novel anatomic variant to the clinical literature. In doing so, this case also reinforces the broader under-recognition of POS in patients presenting with unexplained, posture-dependent hypoxemia.

Embryologic formation of the interatrial septum is a foundational factor in understanding the development of ASDs. Normally, septation begins in the fifth week of gestation with the septum primum extending toward the endocardial cushions, followed by the appearance of the ostium secundum, and then the formation of the septum secundum, which overlaps the septum primum to create the foramen ovale [23]. When this sequence is disrupted, due to incomplete formation or postnatal fusion failure, a persistent interatrial communication may result and, under altered hemodynamic or postural conditions, cause significant right-to-left shunting as seen in POS [24]. This embryological pathway highlights why certain individuals may remain asymptomatic well into adulthood until compensatory mechanisms are disrupted or anatomical relationships shift.

ASDs in the context of POS represent an under-recognized cause of this syndrome. Typically, primum ASDs result from deficient fusion of the septum primum with the endocardial cushions and are anatomically located adjacent to the atrioventricular valves, often accompanied by valve clefts or regurgitation [23]. While secundum ASDs and PFOs are more commonly associated with POS due to their dynamic mobility and favorable location for intermittent shunting, our case demonstrates that even fixed, anatomically distinct primum ASDs can result in posture-sensitive right-to-left shunting under specific structural and hemodynamic conditions [25]. This expands the clinical spectrum of POS beyond its traditional association with more mobile atrial septal abnormalities.

Furthermore, secondary anatomical and physiological contributors play a key role in exacerbating or unmasking shunting through ASDs. In a normal cardiopulmonary system, the left atrial pressure is consistently higher than the right, and thoracic geometry provides a stable configuration that does not predispose to paradoxical flow [26]. However, postural deformities such as kyphoscoliosis, vertebral compression, or elongated thoracic aortas may shift the spatial orientation of the atrial septum, decreasing left atrial pressure while raising right atrial pressure, thereby promoting shunt flow through defects that would otherwise be hemodynamically insignificant [27]. Importantly, these acquired structural changes may not be immediately apparent on routine chest imaging but may emerge with advancing age or thoracic remodeling.

Intracardiac pressure gradients and posture are central factors in the dynamic manifestation of POS. Ordinarily, upright positioning has minimal effect on interatrial gradients in healthy individuals, resulting in the maintenance of left-to-right shunting or no flow through an ASD [28]. However, gravitational shifts and venous pooling during orthostasis may decrease left atrial filling and increase right atrial pressure, temporarily reversing the gradient across a defect and enabling paradoxical embolism or hypoxemia [13]. This effect may be further amplified by exercise, dehydration, or any condition that reduces systemic vascular resistance, exacerbating positional hypoxemia in unsuspected cases.

Recognizing positional hypoxemia is an essential diagnostic factor in identifying POS. Under typical physiological conditions, oxygen saturation remains consistent regardless of body posture, as ventilation-perfusion ratios are tightly regulated [1]. In POS, however, patients experience significant desaturation upon standing, which may be overlooked if oxygen levels are only assessed in the supine position. Clinicians should maintain a high index of suspicion and employ positional oximetry, especially when patients report exertional or postural dyspnea without radiographic or spirometric abnormalities [29]. Positional pulse oximetry can serve as a low-cost screening tool that dramatically increases the yield of diagnostic evaluations.

Imaging modality selection is a critical diagnostic factor in detecting interatrial shunting. Standard practice begins with TTE due to its widespread availability and utility in screening for structural anomalies [30]. However, TTE alone may miss transient or positionally triggered shunts; TEE, especially when combined with agitated saline contrast and performed with positional changes, offers superior sensitivity for identifying small or atypically located ASDs and characterizing flow dynamics [31]. Three-dimensional echocardiography and cardiac MRI can complement TEE when acoustic windows are limited or when multiplanar reconstruction is needed for procedural planning.

Interventional treatment is the defining factor that transforms POS from a chronic syndrome into a curable condition. In general, isolated ASDs or PFOs are managed conservatively if they are not hemodynamically significant, particularly in asymptomatic individuals [32]. However, for POS patients, closure of the interatrial communication, either percutaneously with a septal occluder device or surgically, is often followed by complete resolution of symptoms [33], as was observed in our patient who experienced immediate and sustained normalization of oxygen saturation following surgical repair. Treatment selection should be tailored based on defect type, septal rim adequacy, and the presence of associated cardiac anomalies.

The clinical outcome and diagnostic implications of POS constitute a final point of focus. For most patients with unexplained hypoxemia, cardiac shunts are not routinely considered early in the diagnostic algorithm, leading to significant delays [34]. Our findings emphasize that clinicians should broaden their differential to include fixed structural lesions like primum ASDs, which, though rare, can cause POS under the right anatomical and physiological conditions. Targeted imaging and corrective closure can dramatically improve quality of life and prevent long-term complications of chronic hypoxia [35].

This review has several important limitations. First, our analysis is based entirely on individual case reports, which are subject to publication bias, particularly favoring positive or dramatic outcomes. Second, although we aimed to classify ASD subtypes, 7 of 18 cases did not specify the defect type, limiting our ability to conclusively determine whether secundum ASDs truly dominate the POS spectrum. Third, while we used a structured screening and extraction process, no validated quality assessment tool was applied for the included case reports. Finally, the absence of pooled quantitative data limits the generalizability of findings, and caution should be exercised when extrapolating to broader populations. Nonetheless, the synthesis of these rare cases offers practical diagnostic insight for clinicians managing unexplained positional hypoxemia.

## Conclusions

In current practice, there is no universally accepted diagnostic algorithm for POS, and the threshold for initiating closure varies across institutions. Given the rarity of ASD-associated POS, particularly involving non-secundum subtypes, large-scale studies are unlikely to be feasible. Progress will instead depend on heightened clinical suspicion, systematic positional assessment, and broader awareness of atypical anatomical contributors. Continued detailed reporting of such cases may gradually inform diagnostic frameworks and reduce delays in recognizing this underdiagnosed but highly treatable condition.
